# Acknowledgement of top manuscript reviewers 2014

**DOI:** 10.1186/s12958-015-0002-7

**Published:** 2015-03-10

**Authors:** Antonin Bukovsky

**Affiliations:** The Institute of Biotechnology, Academy of Sciences of the Czech Republic, Prague, Czech Republic

## Abstract

The editor of *Reproductive Biology and Endocrinology* would like to thank all of our reviewers and particularly our top reviewers who have contributed to the journal in Volume 12 (2014).

**Victor Absalon-Medina**

United States of America

**Pilar Alamá Faubel**

Spain

**Erika Alvarenga**

Brazil

**Joao Araujo**

Portugal

**Silvana Arrighi**

Italy

**Kenneth Aston**

United States of America

**Graham Barrell**

New Zealand

**Paul Barriere**

France

**Cordula Bartel**

Austria

**Natalia Basile**

Spain

**Yaakov Bentov**

Canada

**Alexander Beristain**

Canada

**Agnieszka Blitek**

Poland

**Edson Borges**

Brazil

**Leon Brokken**

Sweden

**Theodore Brown**

Canada

**Richard Burney**

United States of America

**Alison Campbell**

United Kingdom

**Bruce Campbell**

United Kingdom

**Koel Chaudhury**

India

**Jerome Check**

United States of America

**Tereza Cindrova-Davies**

United Kingdom

**David Clark**

Canada

**Jacques Cohen**

United States of America

**Laurel Coons**

United States of America

**Robert Cushman**

United States of America

**Ivana Beatrice Da Cruz**

Brazil

**Petros Damos**

Greece

**Jan Danser**

Netherlands

**Tirtha Datta**

India

**Maurizio Dattilo**

Switzerland

**Frank De Jong**

Netherlands

**M L N Deepika**

India

**Gregory Dissen**

United States of America

**Marie-Madeleine Dolmans**

Belgium

**Erling Ekerhovd**

Sweden

**Gloria Evans**

New Zealand

**Kathleen Eyster**

United States of America

**Human Fatemi**

Belgium

**Jens Fedder**

Denmark

**Stephen Ford**

United States of America

**Niamh Forde**

Ireland

**Jason Franasiak**

United States of America

**Anita Franczak**

Poland

**Leyland Fraser**

Poland

**David R. Garris**

United States of America

**Audrey Gaskins**

United States of America

**Francisco Gaytan**

Spain

**Ariane Germeyer**

Germany

**Vince Kornél Grolmusz**

Hungary

**Malgorzata Grzesiak**

Poland

**Roberto Gualtieri**

Italy

**Leah Hawkins**

United States of America

**Paul L Hermonat**

United States of America

**Linda Hong**

United States of America

**Ariel Hourvitz**

Israel

**Monika Kaczmarek**

Poland

**Fusako Kagitani**

Japan

**Hideo Kamomae**

Japan

**Frederick Kan**

Canada

**Suresh Kattera**

Singapore

**Jong-Min Kim**

Korea, South

**Kirstine Kirkegaard**

Denmark

**Kotaro Kitaya**

Japan

**Peter Kovacs**

Hungary

**Sharon Ladyman**

New Zealand

**Susan Laird**

United Kingdom

**Wei Lei**

United States of America

**Sheena Lewis**

United Kingdom

**Harry Lieman**

United States of America

**Yung-Ming Lin**

Taiwan

**Ewa Luszczek-Trojnar**

Poland

**Walid Maalouf**

United Kingdom

**Guido Macchiarelli**

Italy

**Bryce Maciver**

United States of America

**Matthew Mears**

United Kingdom

**Sergey Medvedev**

United States of America

**Roelof Menkveld**

South Africa

**Velja Mijatovic**

Netherlands

**Dean Morbeck**

United States of America

**Neela Mukhopadhaya**

United Kingdom

**Vanessa Murphy**

Australia

**Carolina Nastri**

Brazil

**Krzysztof Nowosielski**

Poland

**Cleida Oliveira**

Brazil

**Johannes Ott**

Austria

**Alessio Paffoni**

Italy

**Rosaura Pérez-Pé**

Spain

**Caitlyn Placek**

United States of America

**Josep V. Planas**

Spain

**Dwi Pujianto**

Indonesia

**Alberto Revelli**

Italy

**Anna Rising**

Sweden

**Fernanda Rocha**

Canada

**Geetanjali Sachdeva**

India

**Marie Saint-Dizier**

France

**Taiju Saito**

Czech Republic

**Smita Salian-Mehta**

United States of America

**Eleonora Salvolini**

Italy

**Daniele Santi**

Italy

**Regiane Santos**

Netherlands

**Heide Schatten**

United States of America

**Dietmar Schlembach**

Germany

**Richard Scott**

United States of America

**Balasubramanian Senthilkumaran**

India

**Ruijin Shao**

Sweden

**Gemma Sharp**

United Kingdom

**Marta Siemieniuch**

Poland

**Petra Sipilä**

Finland

**Mariusz Skoczynski**

Poland

**Berta Soldevila**

Spain

**Tamas Somfai**

Japan

**Astrid Stecher**

Austria

**Xoana Taboada**

Spain

**Toshifumi Takahashi**

Japan

**Alejandro Tapia-Pizarro**

Chile

**Juan J Tarin**

Spain

**Tyl Taylor**

United States of America

**Antoine Torre**

France

**Nam Tran**

United States of America

**Andreas Vernunft**

Germany

**Angela Vinturache**

Canada

**Angel Wang**

China

**Xin Wang**

United States of America

**Camilla Whittington**

Australia

**Izabela Woclawek-Potocka**

Poland

**Chadi Yazbeck**

France

**Marc Yeste**

United Kingdom

**Haiyang Yu**

United States of America

**Ying Zhang**

China

